# Ending the HIV Epidemic in Black America: Qualitative Insights Following COVID-19

**DOI:** 10.1007/s40615-024-01925-1

**Published:** 2024-02-22

**Authors:** Tenesha J. Lewis, R. Patti Herring, Richard E. Chinnock, Anna Nelson

**Affiliations:** 1 School of Public Health, Loma Linda University, 24951 North Circle Drive, Loma Linda, CA 92350 USA; 2 School of Medicine, Loma Linda University, Loma Linda, CA USA

**Keywords:** HIV, COVID-19, Equity, Black Americans

## Abstract

**Background:**

The disproportionate effects of the human immunodeficiency virus (HIV) and the Coronavirus 2019 (COVID-19) on Black American communities highlight structural systems rooted in racism and must be addressed with national strategies that improve both biomedicine and social determinants of health.

**Purpose:**

The purpose of this study was to qualitatively examine the experiences and interpretations of experts in the HIV workforce (local, state, and national HIV-related organizations) regarding the state of HIV and COVID-19 among Black Americans.

**Methods:**

Within key informant interviews and a focus group recorded and transcribed verbatim, fifteen members of the HIV workforce and Black community described their experiences and provided insights to inform ending the negative outcomes resulting from HIV and COVID-19.

**Results:**

Data were analyzed using NVivo software, and eight themes emerged to address disease disproportionality through a Black lens. Themes reflected (1) accessing information and care; (2) key potential partners/stakeholders; (3) investing in Black communities; (4) governmental support; (5) increasing engagement and advocacy; (6) HIV-related community conversations; (7) developments since COVID-19; and (8) the Ending the HIV Epidemic (EHE) trajectory.

**Conclusions:**

Themes directly speak to recommendations to adjust education and policy strategies for HIV and COVID-19 prevention and intervention. Such recommendations, (1) amplifying Black voices, (2) investing sustainable dollars into Black communities, and (3) leaning into advocacy, can bolster the foundation for the HIV workforce and Black community to break ineffective response patterns and lead the fight against these systemic issues of inequity.

## Background

### HIV Progress and Disproportionality

From the earliest days of the human immunodeficiency virus (HIV) and acquired immunodeficiency syndrome (AIDS) over 40 years ago, tremendous advances in biomedicine have been made to address the resultant crisis [1]. Today, medications can help prevent the transmission of HIV and reduce its progression [2]. Remarkably, with the proper adherence to a prescribed medical regimen, the vast majority of people living with HIV (PLWH) are expected to experience lives just as long and full as individuals who do not live with the virus [3]. Further, the risk of transmitting HIV in these cases is typically lower than 1% [4]. Based on the trajectory of research and the development of tools, the near future of HIV may include even more advances, particularly a vaccine and a cure [5].

Despite the progress made in combating HIV, incidence and prevalence rates of the virus continue to be a public health crisis in Black America, identified as all populations of Black people living in the US. Constituting approximately 13% of the US population, Black Americans comprise over 40% of PLWH, as well as just beyond 40% of new HIV diagnoses [6]. These disproportionate effects are particularly evident among individuals identifying as Black gay and bisexual men, as approximately one out of every two Black gay/bisexual men is estimated to acquire HIV during his/their lifetime, as well as Black American women, who are affected by HIV more than women of any other racial or ethnic identity [7]. Further, of all Black PLWH, 85% are aware of their status; however, just under half are retained in care [7].

To address the HIV epidemic and its disproportionality, in 2010, the Obama administration established the first national HIV/AIDS strategy in the US; this plan was primarily aimed at reducing new infections and deaths [8]. This strategy placed some focus on marginalized populations; however, it did not delve deeply into root causes of disproportionality, and the current state of HIV among these populations can serve to demonstrate that the plan did not work as anticipated.

In 2019, the Trump administration presented a framework to end HIV in the US by 2030; Dr. Anthony Fauci revealed the rationale for the Ending the HIV Epidemic (EHE) plan that proposed funding and biomedical strategies [9]. This plan omitted essential information about intersectionality and core social issues, including racism, homophobia, and transphobia, which perpetuate the HIV crisis among Black Americans and other disenfranchised populations [10].

### COVID-19 and Inequity

Parallel to HIV, though a more novel issue, the coronavirus disease 2019 (COVID-19) underscores the systemically racist roots in the US that negatively impact marginalized populations’ health outcomes [11]. At the beginning of the COVID-19 pandemic, states and localities reported shockingly disproportionate rates of mortality among Black Americans [11]. The state of Louisiana, for example, holds a 32% Black American population rate; however, of all COVID-19-related mortalities reported, Black Louisianans comprised more than 50% of deaths at the pandemic’s onset and 36% as of early 2022 [12]. Further, COVID-19 mortality disparities in American localities can be predicted by the proportion of Black residents if the area also possesses inequities in the social determinants of health [11, 12]. Evidently, systemically racist policies mediate Black Americans’ relatively poor COVID-19 health outcomes [11, 12].

The unique susceptibility Black Americans have to health issues like HIV and COVID-19 is known to be rooted in the decades of structural oppression following centuries of chattel slavery; this resultant oppression has led to a plethora of social and structural issues, including poverty, anti-Black racism, housing and food instability, limited educational and employment opportunities, and a diminished quality of mental and physical health [13]. Even though federal, state, and local initiatives and resources are welcome and essential, strategies that solely improve biomedicine without considering social and structural contexts will not sustainably impact the root causes of Black America’s large HIV and COVID-19 susceptibility. Instead, it is critical to respond to disproportionality and gaps in response strategies by exploring how efforts and resources can be pointed toward upstream determinants of health to improve the downstream incidence and prevalence of HIV and COVID-19 among Black Americans.

### Syndemic Interplay Between HIV and COVID-19

COVID-19 and HIV have been identified as a syndemic, or a cluster of issues occurring simultaneously and impacting the same, marginalized populations [14]. Within this syndemic are shared issues regarding systemic racism, poverty, and a lack of high-quality health insurance; COVID-19 is also linked with a lack of safe working conditions. Though PLWH are not at greater risk for COVID-19 infection, HIV may be a significant risk factor for serious negative health outcomes resulting from COVID-19 [15].

Many people impacted by the syndemic live in the US South, the region for eight of the states without a Medicaid expansion [14]. Comparing HIV prevalence with COVID-19 infection data and socioeconomic information has indicated mostly southern counties as “hotspots,” or the areas likely to be greatly impacted by the syndemic. Additionally, greater income inequality in these locations is positively associated with this substantial risk [16].

EHE and COVID-19 responses have caused challenges among providers and community members, including ensuring access to affordable health care [17]. Critical EHE funding has been redirected toward COVID-19 relief efforts [17]. Accordingly, addressing HIV is becoming more difficult, as money is going toward COVID-19 outcomes and may remove much focus away from HIV, since HIV does not affect the entire population as much as it once did [14].

HIV served as a blueprint for how public health professionals responded to COVID-19 regarding testing, contact tracing, and clinical research; moreover, both viruses have severely impacted marginalized populations [15]. In exemplar, new HIV rates continue to rise among people ages 25–34, and there is only 60% viral suppression for Black PLWH [14]. Furthermore, racism and discrimination are repeatedly identified as the roots of great COVID-19 and HIV risk in communities of color; structural changes are indicated as the way to properly address this issue [17]. The underlying societal issues must be addressed, as they exacerbate the negative outcomes associated with both HIV and COVID-19 [14].

### COVID-19’s Influence on HIV Prevention and PLWH

The impact of COVID-19 on PLWH has included negative mental health issues, such as depression, anxiety, and loneliness, as well as social decline, to include changes in sexual activities, income, and education [18]. PLWH, including persons who had the COVID-19 virus, revealed scientific mistrust and an awareness of similarities between stigmas from both COVID-19 and HIV [19]. However, PLWH expressed that acquiring HIV still has had a more significant impact on their lives than acquiring COVID-19, primarily due to the lack of an HIV cure and HIV’s perceived longer term, more permanent and negative, effects on bodies and lifestyles. [19].

Following the onset of the COVID-19 pandemic in the US, 20% of men who have sex with men (MSM) reporting use of pre-exposure prophylaxis (PrEP), or HIV prevention medication, also reported altering their frequency of PrEP usage, and 25% indicated difficulties accessing PrEP and HIV prevention services [20]. Sexual engagement with others was also reported as decreased around the onset of the pandemic and increased in the months following the onset [20]. The COVID-19 pandemic, then, may be connected to an increased risk of acquiring HIV, particularly as a result of reduced access and utilization of prevention modalities, such as HIV testing, PrEP medication, and condoms [20].

### Recommendations from the HIV Workforce

Prior to the COVID-19 pandemic, Lewis et al. [21] uncovered four major themes to address HIV in Black America. The themes were targeted toward (1) reimagining systems, (2) investing in Black communities, (3) universal health care, and (4) leadership development [21]. Each theme was supported with rationale from workforce experts and were summed with policy-based recommendations to implement at the local, state, and federal levels [21]. The study spoke to the needs of Black PLWH and those at risk of acquiring HIV; however, data collection for the research was conducted prior to the onset of COVID-19 in the US. Thus, it is evident that this information must be updated.

### The Social Ecological Model (SEM) in Similar Studies

The social ecological model (SEM) is a theoretical framework that accounts for the interrelatedness among individual, interpersonal, institutional, community, and societal factors [22]. Though the SEM has not yet been leveraged in understanding HIV and COVID-19 through a Black American lens, researchers previously utilized the model in understanding and addressing social barriers related to HIV. The SEM was used to conduct interviews among couples living with HIV to understand barriers around obtaining access to healthcare services [23]. Additionally, several studies used the SEM to conduct interviews for understanding attitudes and behaviors around HIV risk and prevention [24–26]. SEM, then, is an established model in HIV-related research focused on the intersections among society and health.

### Considerations for Effective HIV and COVID-19 Responses

From the aforementioned literature, it is apparent that HIV and COVID-19 disparities were, and continue to be, exacerbated among Black Americans. Since the onset of the COVID-19 pandemic, the HIV workforce is under strain, and at reduced capacity, to navigate prevention and intervention strategies for both HIV and COVID-19. Further, the literature highlights the importance of addressing systems-level issues, such as socioeconomic status, systemic racism, insufficient health insurance, and unsafe working settings, particularly among marginalized populations. However, there is a dearth of literature that explores approaches to address HIV and COVID-19 through a Black lens, indicating the need for increased research in this area. In fact, the lead investigator’s own research [21] must be updated in light of the COVID-19 pandemic, propelling the current study’s design and implementation.

Thus, the purpose of this study was to qualitatively examine the experiences and interpretations of HIV experts regarding the state of HIV and COVID-19 in Black America through the framework of the socioecological model. Based upon themes arising from this examination, we explored recommendations in addressing both HIV and COVID-19 disproportionality through a Black lens.

## Methods

### Study Design

**Fig. 1:**
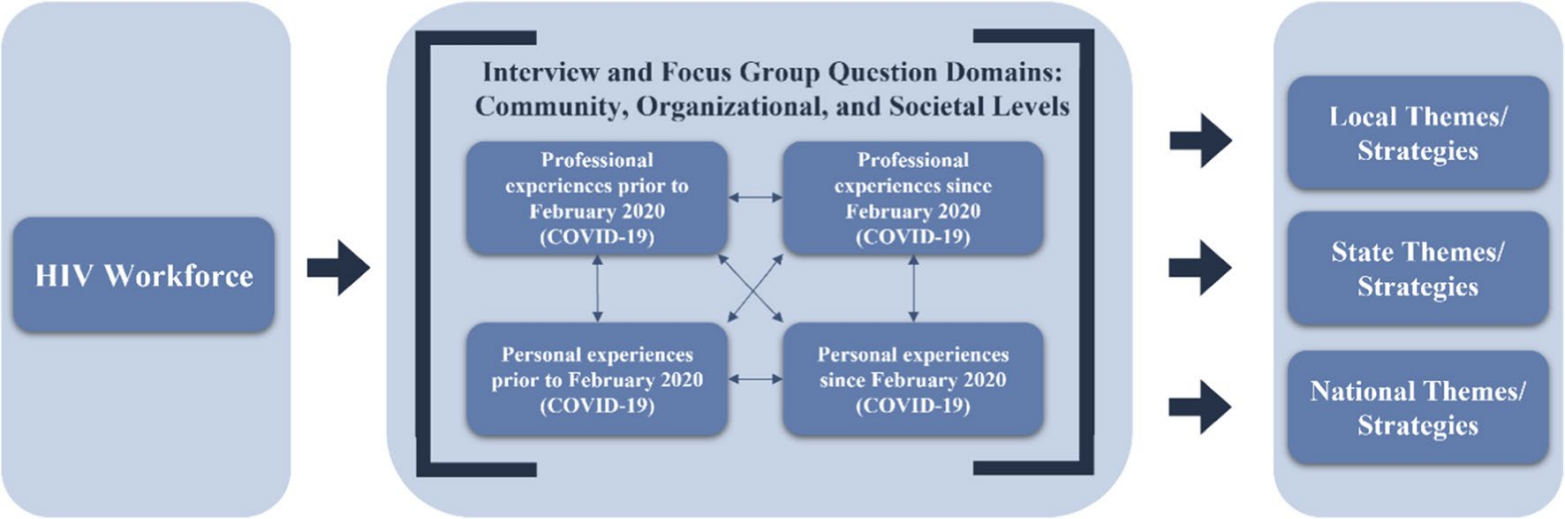
Research framework for the Black HIV workforce qualitative study

Addressing systems-level issues like HIV and COVID-19 requires approaches across various factor levels; accordingly, the SEM guides this research. This study was designed for key members of the HIV workforce to share experiences from their personal and professional lives that speak to prevention and intervention modalities across all SEM levels. Accordingly, the design allowed for emergent themes that point to sustainable measures and recommendations for structural or systemic changes across localities, states, and the nation, as displayed in Fig. 1, which is an original representation of this research, influenced by SEM levels.

The research was also designed along the qualitative approach of phenomenology [27], which leverages data gathered from semi-structured interviews to explore individuals’ lived experiences regarding a common phenomenon. The COVID-19 pandemic’s intersection with HIV was identified as the phenomenon, since the HIV workforce has been forced to navigate outcomes from the two viruses. The study was crafted for the workforce to provide insights from personal and professional lived experiences that have shaped their views regarding effectively addressing HIV and COVID-19 through a Black lens.

Semi-structured questions were created for key informant interviews and a focus group, all to encourage storytelling of participant experiences, challenges, and opportunities regarding the HIV workforce and/or living with HIV, as well as challenges and opportunities in addressing the HIV epidemic in Black America, particularly since the onset of COVID-19. Broadly, the interview and focus group guides allowed for exploration of what has been experienced, how it has been experienced, and the contexts influencing the experiences of this shared phenomenon.

### Study Sample

Participants consisted of the HIV workforce and were all located in geographic areas coinciding with EHE jurisdictions (https://www.cdc.gov/endhiv/jurisdictions.html). These were decision-makers who have worked within the HIV field prior to, during, and since the onset of the COVID-19 pandemic. The first author (T.L.) held an existing connection to credible messengers from the Black community representing HIV boards, organizations, and coalitions across the country at local, state, and national levels. These experts included influential community activists and organizers; HIV care providers; biomedical and public health scientists; national advisory board representatives; PLWH; HIV organization leaders; and community-based organization (CBO) staff.

The inclusion criteria for this study consisted of Black American adults, ages 18 and older, who were able to communicate effectively in the English language and held paid and/or volunteer experience (1) in the HIV field within the US prior to, and since, March 2020, as well as (2) supporting Black Americans at risk of acquiring, or living with, HIV. Potential participants were required to be a local, state, or national level decision-maker, defined as professionally supervising and/or advising others. Individuals were excluded from the study if they did not meet the inclusion criteria or declined audio/video recording.

### Recruitment

Participants were recruited based on purposeful sampling and snowball sampling, and the lead investigator leveraged an extensive and active professional network of over 200 email addresses belonging to individuals presently working in the HIV field at local, state, and national levels. From this, the investigator performed screening against the eligibility criteria and emailed potential participants regarding the study, answering questions, and sending consent forms electronically for review. Additionally, within each recruitment email, potential participants were informed of the eligibility criteria and requested to participate solely if the criteria were met. Participants were recruited until the point of saturation for their diverse perspectives and experiences across local, state, and national levels.

### Data Collection

Upon gaining informed consent, key informant interviews and a small focus group were conducted via the Zoom online platform (https://zoom.us/) to support ease of recording and logistical convenience, as participants resided across the US. The lead investigator who conducted the data collection is a public health equity expert with extensive experience in qualitative research. Accordingly, participants engaged in the study voluntarily and without coercion or influence; they were also automatically provided with mental health support contact information as part of their participation in the study. Study participants could withdraw consent and discontinue participation at any time, for any reason, as well as without reason.

During the data collection process, participants were asked open-ended questions along a semi-structured format on their experiences and insights regarding HIV and COVID-19, including views on key action steps and overall strategies to mitigate, or effectively end, these dual issues among Black Americans. Each key informant interview lasted approximately 45–60 minutes, and the focus group discussion lasted approximately 60 minutes. The interviews and the focus group were recorded to support transcription during data analysis. Audio recording files were transcribed using the Rev transcription service (https://www.rev.com/). The focus group was included as a confirmatory and validating component of the study, intended to explore themes emerging from the key informant interviews. The focus group was conducted following eight key informant interviews, which had undergone transcription and preliminary coding.

### Data Analysis

The researcher responsible for data collection verified and de-identified all transcripts, as well as created the codebook, based on question guides for the key informant interviews and focus group. NVivo 12 Pro for Windows software (https://support.qsrinternational.com/) was used to analyze the resulting data and identify emergent themes and subthemes. The research team, along with an independent external analyst, reconciled themes and subthemes that emerged during the data analysis. The team considered saturation to be reached when no new codes emerged. The codes were then organized into categories, starting with the overall group, followed by subgroups. Finally, the team conducted thematic analyses, which included comparing codes to assess patterns across participant responses.

## Results

For this study, a total of 15 participants were engaged across 11 key informant interviews and one focus group to describe HIV-related experiences in light of the COVID-19 pandemic. An ideal participant number was not established; however, the lead investigator began reaching saturation at three persons and continued data collection with 8 additional interviews and one focus group to support confirmation and validation of findings. Participants were HIV experts, identifying as Black adults, and included cisgender men, as well as cis- and transgender women from the US East, West, South, and Midwest regions. Participants’ HIV work experience ranged from just under one decade to beyond 30 years in support of Black communities as senior/executive leaders, community organizers, program managers, and justice advocates. Table 1 depicts the sociodemographic components of the study participants, sorted by key informant interview and focus group categories.

**Table 1:** Socio-demographics for study participants

Full sample (*N* = 15)		
Age	# KI*	# FG*
18–39	3	2
40 +	8	2
Role		
Executive/senior leader	5	2
Program/project manager	2	2
Community organizer/advocate	4	0
Years of experience		
< 10	1	2
10–19	7	0
20 +	3	2
SEM level		
Local	4	1
State	2	1
National	5	2
Gender identity		
Male	3	0
Female	8	4

^*^KI key informant participants, *FG* focus group participants

Analyzing the key informant interviews resulted in eight recurring themes, further delineated into sub-themes, as indicated in Table 2. Analysis of the focus group discussion confirmed and validated these themes and sub-themes, with no additional themes or sub-themes appearing.

**Table 2:** Thematic code table

Emergent theme	Sub-theme	Quote
Accessing Information and Care (Theme 1)	Providers	“Black community members who have good relationships with healthcare providers will engage [them] about sexual health information.” -P3
	Trusted Community Members	“Our community learns from each other, because there is still this fear or distrust when it comes to science, when it comes to physicians.” -P6
	Media and Messaging	“…Billboards, wraparound messages on the buses…depending on the area you live in, you may see those messages.” -P1
Key Potential Partners/Stakeholders (Theme 2)	Persons and Entities Affiliated with the HIV Field	“Reach out to nonprofit organizations that provide HIV/AIDS services.” -P1
	Persons and Entities Not Directly Affiliated with the HIV Field	“I don’t see people pushing the HIV storyline as much, and there’s new stuff to talk about…Organizations that work to get issues in media should be engaged in making sure they’re talking about HIV.” -P5
Investing in Black Communities (Theme 3)		“Being a Black cisgender woman, I do feel like my population is really being left behind.” -P6
Governmental Support in Addressing HIV Among Black Americans (Theme 4)	Areas Needing Improvement	“I think the government’s largely silent.” -P5
	Areas Where Support is Felt	“I think there’s been more work to try and have federal entities collaborate more.” -P6
Increasing Engagement and Advocacy Across Black America (Theme 5)	HIV as a Community Issue	“People need to see it as their problem or their community’s problem.” -P7
	Inclusion and Acceptance	“…A non-stigmatizing environment…Let’s just talk about all the things…I think that’s finding a way to have that space.” -P13
	Grassroots Activism	“Could there be somebody or a way to sort of encourage that mobilization? Because, given how bad some of it really is, it’s a little surprising we’re not seeing people [protesting] on the streets.” -P7
HIV-Related Community Conversations Before and Since COVID-19 (Theme 6)		“How do we get individuals who are in loving, committed relationships to test?…It’s a very hard challenge…getting them to shift their mind to acknowledge what risk is.” -P15
Developments Since COVID-19 (Theme 7)	Innovations	“Folks who may have challenges taking medication now [have] access to utilizing an injectable.” -P2
	Detrimental Changes	“Coverage through health insurances [is not] moving as fast as the advancements are moving.” -P4
EHE Trajectory (Theme 8)	Current Barriers to Progress	“There needs to be much more diversity, equity, and inclusion in the way that the resources are being sent out, [and] also in the folks who are creating the funding itself.” -P14
	Potential Threats to Progress	“The reasons why HIV impacts Black communities more are…larger structural factors…How do we respond to those structural factors, from housing to substance use to poverty?” -P8
	Potential Opportunities	“…Provide that [testing] service at the library…HBCUs…Those are very important and impactful steps…But also, if something is not working, can we just shift gears completely?” -P15

### Theme One: Accessing Information and Care

Participants explored factors affecting access to HIV prevention and treatment resources. Accordingly, this theme delved into the roles played by healthcare providers, trusted community members, and media-based entities prior to, and since, the onset of COVID-19.

#### Providers

Participants named health departments and community-based organizations (CBOs) as the major providers of HIV prevention and treatment services prior to, and since, the COVID-19 pandemic. As far as Black community members without insurance seeking help, participants identified community clinics or Ryan White clinics as the major places to go, with one key informant saying, “We don’t turn anyone away. We have to see everyone, regardless” (P13). Participants commented that since the pandemic, some clients/patients were lost, and providers are unsure if they sought care elsewhere, dropped out of care, or passed away. Participants noted that many CBOs closed, or paused service, during the onset and initial months of the COVID-19 pandemic.

Participants generally saw the experiences with primary care providers as dependent on the relationship, in that the care provided has been significant, but, as one informant added, “I don’t think…it’s always culturally sensitive or relevant” (P8). Regarding primary care physicians/clinicians, another key informant noted,

A lot of the community-based organizations…were closed down during the pandemic, so folks had to find other places to go. I think they really started getting them from primary care physicians. (P5)

#### Trusted Community Members

Participants commented on an understandable, existing state of general mistrust among Black Americans regarding scientific and medical professionals. In these cases, participants noted that trusted persons from the community are key sources of information, and from the community members’ perspectives, there is likely action based on what one key informant described as “*…*what I hear…from family, friends—so people that I trust” (P2). Family, friends, clergy, and even academic professionals were said to be sought out for HIV-related resources and details.

#### Media and Messaging

When discussing the ways in which information is received around HIV prevention and treatment, participants responded that advertisements (e.g., billboards, commercials) and the internet/social media were major sources, particularly following the onset of the COVID-19 pandemic, when in-person access was limited. A key informant commented,

I think it got a little harder, because people were kind of quarantined…so things shifted to online…As things shifted to online, some people were left out and they didn’t have that information. (P4)

Another key informant noted,

Some people would also look up information online. I know online resources tend to still be…a source of information for many of us, particularly those of us on the younger end of the scale. (P3)

### Theme Two: Key Potential Partners/Stakeholders

Across the interviews and focus group, participants underscored the importance of a broad and inclusive partnership approach to drive positive change around HIV in Black America. Participants emphasized engaging individuals and entities inside and outside of the healthcare sector; these included influential figures, educational institutions, businesses, and policymakers. Collaborative efforts were highlighted as a means to raise public awareness and address health issues effectively.

#### Persons and Entities Affiliated with the HIV Field

Participants considered people and entities who should be engaged in conversations, planning, and action around addressing HIV among Black Americans, and they indicated nonprofits providing HIV/AIDS services, health departments in rural areas, physicians, and case managers should all be involved. Of particular interest were the priority populations themselves, including people successfully utilizing PrEP and PLWH. An interview participant commented, “I think it’s a huge thing when you have individuals that are out living a whole, full, and happy life with HIV” (P4).

#### Persons and Entities Not Directly Affiliated with the HIV Field

Most participants indicated the importance of having social influencers (e.g., Shannon Sharpe, Lil Nas X, Billy Porter) frequently communicating accurate HIV information in public. In the focus group, one person commented, “I would…try and get people with influence to talk about the subject” (P9), while another added, “Lil Nas X…used his celebrity status to raise money for a lot of CBOs and incorporated [the topic of HIV] into his show [at the Video Music Awards]” (P11).

Colleges, K-12 schools, Black-led businesses from the private sector, persons affiliated with the media, and policymakers were also cited as important to mobilize, primarily for their ability to connect with people in spaces that could lead to sustainable change. In fact, one key informant brainstormed around major initiatives coming from large institutions, including.

…out of the White House…It has to be something large scale that gets people’s attention. Everything that’s done in this field is done behind the scenes, so the public doesn’t really get to see it. (P5)

### Theme Three: Investing in Black Communities

Participants in the key informant interviews and focus group wanted to make it clear that all Black people need resources and support. They pointed out the importance of considering Black people born in the US, as well as Black people who immigrate to this country. Participants noted that Black people born across the African diaspora all need support, to include Black people born in Africa and in the Caribbean. According to a key informant, “Black immigrants are often left out” (P5) of conversations around support, particularly when considering HIV.

Participants indicated that priority populations include Black trans and cis women, as well as Black trans and cis men. A focus group participant commented, “Black trans men get left out of the conversation” (P11). Of the cis men, participants remarked that MSM and men who have sex with women should be considered. One key informant added, “I’ve done a lot of work with Black men who have had sex with [both] men and women…and are at risk for HIV” (P7).

Moreover, the participants felt that older generations (persons over 50 years old) and younger generations (persons under 25 years old) of Black people can also benefit from additional, specific resources. Participants felt that age-based perceptions need to shift, with a key informant saying, “We’re talking about sexual health as if sex stops at 50” (P2). From the participants’ standpoint, babies affected by vertical transmission, meaning being born with HIV, do not receive enough attention, and it is important to know that growth from infancy still counts as aging. One key informant added, “We don’t consider [populations affected by vertical transmission] aging with HIV, [because] when we hear aging, we think of people who are older…but everybody’s aging every day” (P6).

Participants also elevated the people who use substances, have disabilities, and live in rural communities, as well as pregnant people and people without secure housing, food, and/or transportation. Ultimately, the consensus across key informant interviews and the focus group was that, even though it is important to tailor messaging to specialized groups of people within the Black population, it is equally important to consider the population at large and know that by the nature of being Black, people are already disenfranchised and under-resourced.

### Theme Four: Governmental Support in Addressing HIV Among Black Americans

When discussing the government, participants generally emphasized the need for improved support, while also acknowledging pockets of progress and representation. There were mixed experiences around the government’s involvement in HIV initiatives. Many participants expressed overall dissatisfaction, citing disruptions related to the COVID-19 response. PrEP promotion at the local level, increased federal collaboration, and involvement from Black decision-makers were all seen as positive steps toward understanding community-level needs and advocacy.

#### Areas Needing Improvement

Overwhelmingly, the majority of participants from the interviews and focus group reported their government-related HIV experiences more negatively than positively. Multiple key informant interviews echoed reactions of a “lukewarm and diluted” (P8), limited, and primarily silent local, state, and federal government. Additionally, participants across the study reported either distrust toward, or unreliability from, the federal government. Furthermore, participants frequently discussed how HIV plans were derailed with the onset of COVID-19, with focus group participants saying, “It feels like there wasn’t room to both talk about COVID and HIV” (P12), and “I think there was a lot of fanfare and excitement before COVID, but I definitely think that COVID sucked the air out of a lot of this” (P11).

The federal government’s work was regarded as not very visible, with too much bureaucracy and not enough support around the Director of the Office of National AIDS policy; several participants remarked that, outside of this Director, much of the higher federal and state leadership cannot relate to issues on the ground. One key informant added,

My city councilperson…she’s been very visible…Me seeing someone like her continuing this kind of conversation…would be powerful…It’d be great for [President Biden] to have a message, but I don’t think that would be as impactful as that local face. (P13)

Another key informant noted that the Minority AIDS Initiative was expected to prioritize organizations consistently led by people of color and “in a few years, that was diluted” (P8) to now aiding organizations that only work with, or support, marginalized populations. The informant indicated the organizations receiving regular support from the Initiative are not typically led by people of color.

From a solutions perspective, participants offered areas where government sectors could immediately begin to improve. Of note, was funding trusted community members and organizations doing the work and not just entities that are popular or established. Also, one focus group participant commented that providing capacity building programs, such as programming on grant writing, to community members would be helpful. Several participants elevated a need for better support of the HIV workforce, which has been impacted by COVID-19 in both professional work and among personal lives, to include losing loved ones and experiencing mental health strain. Further, a commonality across most interviews and the focus group was the negative impact of Medicaid not being expanded in all states, with a key informant stating,

I think about…some of the southern states who opted not to expand Medicaid and how that impacted largely Black and Brown people who are lower income or poor, and what does this do for their health outcomes and the health outcomes of their families? (P3)

Participants commented on the need to hold elected officials accountable, as well as the need to vote. One key informant said, “When we work at HIV, we also have to work in voter education and voting registration, because who you vote for matters and affects your healthcare and access” (P5). Another remarked,

I think it’s really important for us to be engaged in the politics of our local communities and recognize that these people are…being paid by taxpayer dollars, and they’re responsible and accountable to our needs. (P3)

#### Areas Where Support Is Felt

Regarding advancements across the government, participants stated that localities continued to promote PrEP, and federally, there was additional funding and more intentional collaboration across entities since the COVID-19 pandemic. Key informant interview participants noted that recent years had seen more representation in government, including the Director of the Office of National AIDS Policy—a Black PLWH who “understands community” (P8) and how community-level work is done. One informant commented,

We depend on [the diverse representation in government], because they have that key access to speak up for us and create a space so that we can come in and speak up, as well. (P6)

### Theme Five: Increasing Engagement and Advocacy Across Black America

When considering how to encourage Black Americans to engage and advocate around HIV, participants called for collective community action to address HIV as a social justice issue and connected HIV to broader health disparities driven by social and structural determinants. Faith leaders and communities were identified as important in driving change, with a recognition that some stigma still exists. Participants also called for a shift in how HIV information is communicated to foster inclusion and acceptance within the Black community. Grassroots activism, especially among young people, was seen as a potential catalyst for increased advocacy alongside other social justice issues to amplify rights and awareness.

#### HIV as a Community Issue

People in the Black community regularly come together to advocate for change, and participants across the key informant interviews and focus group called for this type of effort regarding HIV, establishing this health matter as a social justice issue, alongside other health outcomes impacted by social and structural determinants of health. As one key informant stated, “It requires the Black community as a whole to look at this issue as a community issue and not an individual issue” (P3).

#### Inclusion and Acceptance

Participants noted that faith leaders and faith communities need more engagement, as the Black Church has always been one of the strongest pillars in the Black community. They commented that if HIV needs to be addressed from the collective community, it would be logical to believe the Church would be pivotal in spearheading some of this change. Participants often noted that a problem arises when Black people associate HIV with a “type of person” who may not be in alignment with their beliefs and attitudes, with one key informant saying, “I think [HIV is] still considered a taboo subject, especially in the Black Church. It’s kind of like, ‘Yes, we love you, but you’re living wrong’” (P4). The participants shared that real change and support from Black people, particularly those within faith communities, means acceptance of, and advocacy for, *everyone* in the Black community.

One common request across all participants was for society to adjust the way in which HIV information is communicated. Participants generally wanted to acknowledge the progress already made across 40 + years of work, while the efforts continue to improve. They noted a need for ideological changes, including not believing “those people” acquire HIV, but to include and accept “our people.” Participants across the study also discussed the need for safe community spaces that are not centered on HIV but centered on meeting people’s needs or just for having fun, with an informant adding, “One of the bigger things I think is creating a safe space for individuals to feel as though they’re accepted and can live their lives openly and free” (P4).

#### Grassroots Activism

When asked what could increase Black advocacy around HIV, participants commented on the importance of physically attending events and speaking up about needs. Several participants pondered a grassroots movement spearheaded by young people, including wondering if there could be a movement from within ballroom culture. One focus group participant said, “Oftentimes, the youth are the ones with the momentum” (P10), while a key informant remarked,

Why aren’t more people on the streets about Medicaid expansion, or reproductive rights, or them sending the money back from Tennessee? Or these drag show bans, et cetera?…There could be a young people’s movement that really was just lifting up…rights. (P7)

### Theme Six: HIV-Related Community Conversations Before and Since COVID-19

HIV has influenced community mistrust and skepticism around the COVID-19 response; participants discussed the importance of improving HIV understanding and relatability at a community level. A few participants remarked that many community members recall the 40 + years of HIV research without a vaccine or cure and tend to compare that to the brief period of time to develop a COVID-19 vaccine. Participants also alluded to many Black Americans not seeing HIV as a current problem or not being aware of the advancements in HIV prevention and treatment, such as the U = U movement (https://preventionaccess.org). From this, one interview participant replied, “I think for many people in the Black community, they don’t see themselves as vulnerable to HIV” (P3). Within the interviews and focus group, another issue around current conversations in Black spaces dealt with relatability. Participants indicated that Black women were not able to relate to the messaging around HIV, and a key informant stated,

A lot of women that I came in contact with…[were like], ‘I don’t really feel like I can relate to it’…A lot of women…[were] feeling very discouraged and thinking ‘Maybe there are no services for me’. (P1)

Participants generally felt that COVID-19 has helped connect concepts of inequity with disease outcomes, potentially helping to reshape society’s perception of HIV and the social and structural determinants of health. One interview participant responded,

I think that, post-COVID, there’s a bit more of an understanding of how health disparities impact Black communities…There’s not complete understanding, but I think that COVID showed there are all of these other issues at play that are outside of behavior and drive how diseases impact Black communities. (P8)

### Theme Seven: Developments Since COVID-19

Participants generally expressed positivity about recent advancements, including lessons from COVID-19, innovative prevention strategies, increased messaging, telehealth expansion, and injectable prevention and treatment. However, frustration was voiced over the lack of a national PrEP plan, harmful budget and policy changes, and staff burnout, aggravated by the toll of the COVID-19 pandemic on mental health.

#### Innovations

The participants generally felt positively around the advancements in HIV since the onset of COVID-19. They expressed that some of the lessons learned from HIV had been used in the COVID-19 response and vice versa; one example was given by an interview participant, who cited “mRNA technology and [research for] HIV vaccine development” (P3). A focus group participant elevated an innovation around leveraging the 10- to 15-minute waiting periods following COVID-19 vaccinations at clinics, saying, “I’ve heard about efforts to use that time to try to connect with people around HIV testing or PrEP initiation” (P11). Recent developments mentioned also included presidential support for a national PrEP plan; an increase in HIV messaging, particularly around PrEP; an increase in telehealth services and the availability of self-test kits; and the rollout of injectable prevention and treatment medications—something that the participants perceived as critical to the fight against HIV. Participants discussed the usefulness of bimonthly and semiannual injections, with a key informant commenting how this would support priority populations, saying,

[The injections would help] people who may be struggling with adherence to know, ‘Six times a year, versus 365 days, I’ve got to be reminded, or prioritize my medication for HIV prevention’. (P2)

#### Detrimental Changes

Multiple participants reported frustration around changes that occurred since the onset of COVID-19. Key informants commented that “Congress didn’t approve the President’s budget…for a national PrEP program” (P8), as well as “There have been budget cuts…which have taken away the different programs within the community” (P4). One key informant noted, “Roe versus Wade was overturned, which is challenging…Truthfully, Planned Parenthood and similar institutions tend to be more culturally responsive than a lot of other institutions for LGBTQ people, particularly trans persons” (P3).

Another interview participant discussed burnout and how the death surrounding workforce members during the height of the COVID-19 pandemic took a major toll on providers’ mental health, saying,

I’ll never forget…it was really rough working…because there [were] just a lot of memorials—more than often…It was like every week there was a different person, a different patient. A lot of them were HIV positive…lots of death, lots of grief; staff still having to show up every day was really challenging. (P13)

### Theme Eight: EHE Trajectory

Participants explored the progress, barriers, and prospects related to EHE goals, particularly for Black populations. There were expressed concerns about the slowing momentum and reduced focus on HIV messaging during the COVID-19 pandemic. Participants also highlighted issues with equitable data collection and continued stigma. Repeatedly, participants emphasized addressing social and structural determinants of health alongside biomedical advancements, calling for culturally relevant messaging, antiracist training, and integrated health and wellness approaches.

#### Current Barriers to Progress

Various participant-expressed frustrations indicated decreased momentum and progress since the national rollout of EHE goals. Some noted that HIV-related messaging has been placed on the backburner since COVID-19; a key informant added, “We still have people testing positive for HIV every day” (P1). Additionally, one focus group participant commented on data collection not accurately accounting for non-binary identities or intersectionality, saying,

Data collection is still really exclusionary in a lot of ways…like the way in which trans folks are counted is often inaccurate. I don’t think people even have an idea half of the time of how to account for non-binary individuals…If we don’t have an accurate count, then likely those areas are underfunded and just generally underserved. (P12)

Participants also generally expressed that some lessons learned from HIV were not fully applied during the COVID-19 pandemic, including that many entities are focused on disease, instead of health, as well as that there is still stigma and disbelief of people when they discuss their experiences and behaviors. One key informant stated,

[If] a Black man would be diagnosed with HIV and he identified as heterosexual, more often than not the conversation was he wasn’t telling the truth…With that type of stigma, why would I want to go into a health department to learn my HIV status? (P2)

#### Potential Threats to Progress

The participants generally indicated concern when discussing the trajectory of ending the HIV epidemic among Black Americans and wondered what would happen when the epidemic is over among non-Black populations. According to participants, the effective end of the HIV epidemic will not be reached for Black Americans by 2030, and this was understood even prior to COVID-19 occurring. A key informant added,

Before COVID, those of us Black people in this HIV space already knew we would not get to the end of the epidemic by 2030…With everything that’s happened with COVID, we may be looking into 2050…before we can declare victory for HIV in Black communities. And quite honestly, that is my fear…of what happens when white people get to declare that HIV is over for them. (P2)

General thoughts were that it has been over 40 years without an HIV vaccine or cure, and ultimately, biomedicine alone will not end the HIV epidemic; social and structural determinants of health need to be addressed, as well. One interview participant commented, “It’s been over 40 years and really we should not still be fighting as hard as we are, but this is where we are, and this is where we’re going to stay until something is done better” (P6). Another stated,

I think that PrEP is a great example of how you can invest all this money in the development of innovative biomedical tools, and it doesn’t matter if there’s all these other issues that are [not] responded to… in a way that’s honestly equitable. (P8)

#### Potential Opportunities

In considering the trajectory to end the HIV epidemic among Black populations, participants noted opportunities for flexible programming and to incorporate HIV as a general part of health and wellness. Largely, this involved increasing culturally relevant messaging across the general public and sustainably weaving antiracist training and practices into everyday work. One focus group participant commented,

As someone who did capacity building…for very white [spaces], usually health departments, it would be like a one-off training…no more than like 20 hours, and that would just be that, and there would never be a request or a desire for any kind of follow up. (P12)

Another added,

A lot of [health issues] come down to the same social determinants of health really impacting them…[combine] them with needs for more substantial housing or transportation…not just taking as much of a siloed approach… only about HIV. (P11)

## Discussion

This study was an exploration of HIV in Black America following over 40 years since the onset of the crisis and after 3 years of a global COVID-19 pandemic. Leveraging the SEM as the research’s framework, it qualitatively examined the experiences and interpretations of HIV experts regarding the state of HIV and COVID-19 in Black America and explored how the COVID-19 pandemic impacted the HIV workforce’s everyday efforts to address community, organizational, and societal inequities among Black Americans. It also worked to determine how the COVID-19 pandemic has affected the trajectory of the national initiative to effectively end HIV by 2030. Themes were identified to recommend next steps for addressing HIV and COVID-19 disproportionality through a Black lens. Many of the themes discussed included participants’ ideas for improvements that can be made. The participants’ commentary, then, can be summed into the following broad recommendations for action:

1. Actively engage individuals and organizations affiliated with HIV, as well as those without any direct affiliation, to create space and amplify the voices of Black Americans. This includes incorporating sustainable equity training programs and policies across health agencies, as well as connecting with the media to portray culturally relevant information on television programs, movies, and advertisements. Findings from Themes One, Two, and Eight involve convening stakeholders, collaborating across disciplines, and ensuring Black voices are in decision-making spaces. In other literature, some of the populations (e.g., cisgender Black women) most affected by HIV reported not seeing themselves relevantly represented in media and marketing and could benefit from intentional engagement [28]. Also, health agencies that incorporate equity-based strategies, like trainings and storytelling, have been shown to increase providers’ understanding and culturally relevant care [29]. Amplifying Black voices is critical toward accurate portraying, and power sharing, with people disproportionately impacted by HIV.
2. Invest sustainable public/government and private dollars into Black communities across the US to support all Black people, who are disenfranchised by inequitable systems and structures. Across Themes Three, Four, and Seven, findings show it is important to address the root causes of health issues like HIV, and not solely treat the outcomes themselves. This recommendation is supported by evidence that structural racism has had centuries to become institutionalized within this country, and an equity-focused and community-driven approach to sustainable collaboration, data collection, and data usage across governmental entities is critical [30].
3. Lean into advocacy for HIV-related change. Findings from Themes Five and Six indicate this can involve looking inward at biases and misconceptions, working to shift internal perspectives to better align with facts about who is at risk of acquiring HIV and how prevention and treatment modalities function. It can also involve mobilizing people to address relevant issues, such as voting and injustices across localities, states, and the country. Research shows critical awareness of health injustices, self-reflection, community mobilization, and consciousness on behalf of the community are all key tenets that can be leveraged toward HIV-related activism and change [31].

In sum, truly receiving, processing, and acting upon the insights and experiences of the HIV workforce is essential toward achieving long-term, sustainable change and effectively ending the HIV epidemic among Black Americans.

### Limitations and Considerations

Like other efforts that address equity-based health outcomes, this research is subject to several limitations. Although study participants span diverse geographies, as well as lived and work experiences, selection bias could exist. The Black American population is not monolithic; thus, participants may not reflect the fullness of views within the Black HIV workforce. From the initial network of over 200 people, 30 individuals were contacted; however, due to scheduling constraints within the month of data collection, not all were able to participate. Accordingly, the study’s methodology could have been supported with the inclusion of additional focus groups across a lengthier period of time. Moreover, as a consideration, future research may benefit from the additional statistical analysis structure that mixed methods techniques could offer.

The commentary and recommendations iterated within this study also assume openness to change from public and private sectors across localities, states, and the nation. Progress toward addressing the issues described could be deterred if any one of these sectors is not in alignment with the path forward. Additionally, because the indicated needs are systemic, solutions are wrought with potential challenges caused by various confounding factors. A set of strategic plans with indicators at the national, state, and local levels would support progress assessment as efforts to eradicate the HIV epidemic proceed.

Finally, the lead investigator’s (T.L.) embraced identity as a cisgender Black American woman from the rural South all shape the lens through which this study has been crafted and implemented. Accordingly, this research is not intended to be fully without the rich, bold, and unapologetic personal insights of those involved; instead, both “head and heart” were woven into this work, modeling a modern approach to human-driven health equity efforts.

## Conclusion

Despite any limitations, findings from this research can positively affect policies and best practices in addressing HIV and COVID-19 disproportionality. Study outcomes were intended to support marginalized populations seeking justice for an insufficient societal response to the detrimental effects of the two viruses. The concepts and needs detailed within this study are ambitious, requiring a great deal of collaboration across disciplines and at the local, state, and national levels. However, findings are derived from scientific evidence and the HIV workforce’s lived and work experiences following over four decades of efforts to address HIV and the years of a global COVID-19 pandemic. Study outcomes from this research contribute toward (1) building a platform for the HIV workforce and the Black community to own the fight against the dual viruses and (2) offering education on how best to address disparities directly resulting from ineffective systems-level responses to these socially influenced health issues.

## Data Availability

The participants of this study did not give written consent for their data to be shared publicly.
