# Correction: Ending the HIV Epidemic in Black America: Qualitative Insights Following COVID-19

**DOI:** 10.1007/s40615-024-01974-6

**Published:** 2024-03-19

**Authors:** Tenesha J. Lewis, R. Patti Herring, Richard E. Chinnock, Anna Nelson

**Affiliations:** 1 School of Public Health, Loma Linda University, 24951 North Circle Drive, Loma Linda, CA 92350 USA; 2 School of Medicine, Loma Linda University, Loma Linda, CA USA

**Correction: Journal of Racial and Ethnic Health Disparities**

10.1007/s40615-024-01925-1

The state in author affiliation 2 for this article was incorrectly given as VA (rather than CA) in this article as originally published.

Also, “Black” in the caption for Fig. 1 was incorrectly rendered with an initial lower-case letter.

The original article has been corrected.

